# Hidden diversity in Senegalese bats and associated findings in the systematics of the family Vespertilionidae

**DOI:** 10.1186/1742-9994-10-48

**Published:** 2013-08-12

**Authors:** Darina Koubínová, Nancy Irwin, Pavel Hulva, Petr Koubek, Jan Zima

**Affiliations:** 1 Department of Zoology, Faculty of Science, Charles University, Viničná 7, 12844 Praha 2, Czech Republic; 2 Biology Department, University of York, Heslington, YO10 5DD York, UK; 3 Institute of Vertebrate Biology, Academy of Sciences of the Czech Republic, Květná 8, 60365 Brno, Czech Republic; 4 Department of Forest Protection and Game Management, Faculty of Forestry and Wood Sciences, Czech University of Life Sciences, Kamýcká 1176, 16521 Praha-Suchdol, Czech Republic

**Keywords:** Vespertilionidae, Systematics, Phylogenetics, DNA, Karyotypes, Western Africa

## Abstract

**Introduction:**

The Vespertilionidae is the largest family of bats, characterized by high occurrence of morphologically convergent groups, which impedes the study of their evolutionary history. The situation is even more complicated in the tropics, where certain regions remain under-sampled.

**Results:**

Two hundred and thirteen vespertilionid bats from Senegal (West Africa) were studied with the use of non-differentially stained karyotypes and multi-locus sequence data analysed with maximum likelihood and Bayesian methods. These bats were identified as 10 different taxa, five of which were distinctive from their nominate species (*Pipistrellus hesperidus*, *Nycticeinops schlieffenii*, *Scotoecus hirundo*, *Neoromicia nana* and *N*. *somalica*), based on both karyotypes and molecular data. These five cryptic taxa are unrelated, suggesting that these West African populations have long been isolated from other African regions. Additionally, we phylogenetically analysed 166 vespertilionid taxa from localities worldwide using GenBank data (some 80% of the genera of the family) and 14 representatives of closely related groups, together with our Senegalese specimens. The systematic position of several taxa differed from previous studies and the tribes Pipistrellini and Vespertilionini were redefined. The African *Pipistrellus rueppellii* was basal to the *Pipistrellus*/*Nyctalus* clade and the Oriental species *Glischropus tylopus* was basal to the East Asian pipistrelles within the tribe Pipistrellini. The African genus *Neoromicia* was confirmed to be diphyletic. Based on GenBank data, *Eptesicus* was polyphyletic, with the Asian *E*. *nasutus* and *E*. *dimissus* both supported as phylogenetically distinct from the *Eptesicus* clade. The subfamily Scotophilinae was confirmed as one of the basal branches of Vespertilionidae.

**Conclusions:**

New taxa and new systematic arrangements show that there is still much to resolve in the vespertilionids and that West Africa is a biogeographic hotspot with more diversity to be discovered.

## Introduction

The vespertilionid bats form the largest chiropteran family with approximately 48 genera and 407 species [1]. The representatives of the family are found worldwide, with the highest diversity in the tropics [1]. Due to the occurrence of convergent, parallel or mosaic evolution and the retention of plesiomorphic conditions in several vespertilionid groups, it is difficult to determine the generic status and estimate phylogenetic relationships of many species. Until recently, the phylogenetic relationships of vespertilionid bats were studied mainly through analyses of traditional morphological [2,3] or cytogenetic characters (reviewed in [4-6]), but molecular phylogenetic methods using DNA sequencing have been extensively applied and are changing many traditionally recognized groups (e.g. [7-9]). Some genera have been separated into distinct families (Miniopteridae, Cistugonidae), several tribes were reorganized or even rejected, and a number of cryptic species have also been discovered (e.g. [7-14]). Five subfamilies are currently recognised within Vespertilionidae (Vespertilioninae, Myotinae, Antrozoinae, Murininae, Kerivoulinae and Scotophilinae [1,15]), but the number varies from four to eight according to different authors. Based on morphology, the subfamily Vespertilioninae was traditionally divided into the tribes Lasiurini, Nycticeiini, Pipistrellini, Plecotini and Myotini [16]. Later, analysis of bacular morphology excluded Scotophilini [17], karyotypes separated Eptesicini [5], with other tribes being Vespertilionini, Nyctophylini and Antrozoini [10]. The genera belonging to these tribes changed their systematic position frequently. Monophyly was supported by karyotypes and studies of mitochondrial DNA only for Lasiurini, Scotophilini and Antrozoini; *Myotis* was placed in its own subfamily [5,7,10]. Based on morphology, the tribe Nycticeiini was thought to include *Otonycteris*, *Rhogeesa*, *Baeodon*, *Scotomanes*, *Scotoecus*, *Scoteinus*, *Scotophilus* and *Nycticeius*[16]; however, molecular studies showed a different composition: *Glauconycteris*, *Lasionycteris*, *Nycticeius*, *Scotomanes*, *Eptesicus* and *Arielulus*[7,9,10]. However, *Nycticeius* position was unresolved with nuclear markers and opened a discussion regarding the appropriateness of the tribal name if it was removed from the group [9,10]. Recently, nuclear markers indicated the existence of “perimyotine“ (*Parastrellus* and *Perimyotis*) and “hypsugine“ (*Chalinolobus*, *Hypsugo*, *Laephotis*, *Neoromicia*, *Nycticeinops*, *Tylonycteris* and *Vespadelus*) groups within Vespertilioninae [10]. The tribe Pipistrellini has also been revised extensively. The tribe was thought to contain *Pipistrellus*, *Glischropus*, *Scotozous*, *Nyctalus* (supported by karyotypes [5]) and *Scotoecus* (based on molecular markers [7]). The bacular morphology delimited the Vespertilionini as: *Eptesicus*, *Glauconycteris*, *Histiotus*, *Ia*, *Mimetillus*, *Tylonycteris* and *Vespertilio*[17]. Later, mitochondrial DNA analysis confirmed inclusion of *Hypsugo* and *Neoromicia* (previously belonging to *Pipistrellus*[17]), while excluding others, e.g. *Eptesicus*[9]. The genus *Scotophilus* was traditionally classified within the tribe Nycticeiini [1,2], but has also been placed into Plecotini [18] or Eptesicini [19]. However, bacular morphology [17], mitochondrial DNA [7] and cytogenetic analyses [20] supported the separation of *Scotophilus* to a distinct tribe Scotophilini. The tribe was elevated to a subfamily rank (Scotophilinae [15,21]) and even proposed to be renamed as Philisinae, based on shared characteristics with the extinct genus *Philisis*[21].

The systematics of the family Vespertilionidae, especially of some groups, is therefore extremely complicated (see the brief overview of the classification history of the vespertilionids [10]). Even in regions where research effort is extensive, the relationships of many groups still remain unclear. The generic status of the American pipistrelles was clarified only recently [22] and new cryptic species are still being discovered in Europe and Asia (e.g. [23,24]). This implies that in other regions, such as tropical Africa, where large geographic areas are under-sampled, the systematic classification of vespertilionid bats requires assessment and the evolutionary relationships between species are even less certain.

In Africa, 105 species of vespertilionid bats belonging to 16 genera are currently recognized, thus representing approximately 25% of the diversity of the family [15]. Despite the long history of zoological surveys on the African continent, knowledge of the bat fauna is patchy and usually targeted to particular regions. Many vespertilionid species lack external taxonomically informative characters and are therefore difficult to identify using morphological keys. Consequently, the possibility of adding new distribution records or even discovering new species is still high [11,25-27].

Western Africa is considered one of the biodiversity hotspots [28]. Senegalese vespertilionids are currently thought to number 50 species [1,15,29-34]; however, this is still likely to be an underestimation. We assessed the diversity of the local vespertilionid bat fauna, focused mainly on the relatively small area (approximately 9,130 sq km) of the National Park Niokolo-Koba (inscribed on the List of World Heritage in Danger). Our aim was to identify the species and to understand their systematic relationships within the family. We used a multi-locus approach, sequencing two nuclear (recombination activating gene 1 and 2 – *rag1* and *rag2*) and six mitochondrial genes (cytochrome *b* – *cytb*, *tRNA*^*Thr*^, *12S*, *tRNA*^*Val*^, *16S* and NADH dehydrogenase subunit 1 – *nd1*). We chose these genes as 1) they are presumed to be informative at different depths in the tree (given their different rates of evolution) and 2) allowed us to make use of previously published sequences of related bat species. These data were analysed with Bayesian analyses (BA) and Maximum Likelihood (ML) methods for inferring phylogeny. To obtain another independent assessment of taxonomic status, we also examined standard non-differentially stained karyotypes from selected specimens.

We identified ten currently recognized species in a sample of 213 bat specimens from Senegal and found five cryptic taxa closely allied to *Pipistrellus hesperidus*, *Nycticeinops schlieffenii*, *Scotoecus hirundo*, *Neoromicia nana* and *N*. *somalica* based on molecular systematics and karyotypes. Our findings, based on the Senegalese material and GenBank data of worldwide origin, clarified the phylogenetic position of the African *Pipistrellus rueppellii* and Asian *Eptesicus dimissus*, *E*. *nasutus* and *Glischropus tylopus*. We found support for different compositions of the tribes Pipistrellini and Vespertilionini compared to recent publications (see overview in [9]). The tribe Scotophilini was recovered with support as the second most basal branch within the Vespertilionidae and the African genus *Neoromicia* was confirmed to be diphyletic.

## Results

### Cytogenetic analysis

**Figure 1:**
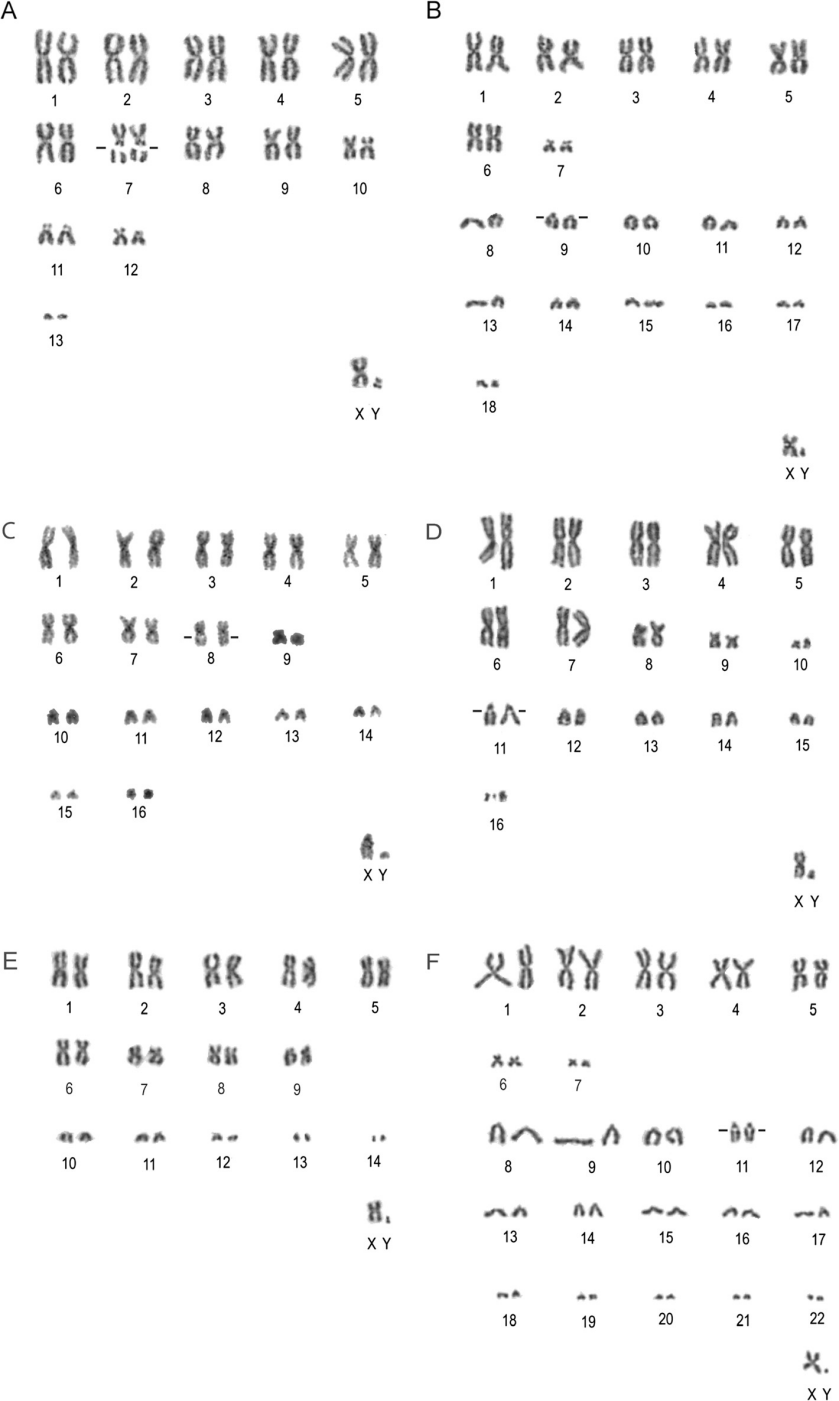
**Male karyotypes of the vespertilionid species studied. A** – *Neoromicia somalica*, IVB S1209 (secondary constriction on the seventh pair of chromosomes); **B** – *N*. *rendalli*, IVB S1212 (secondary constriction on one pair of acrocentric chromosomes – number nine); **C** – *N*. *nana*, IVB S1210 (secondary constriction on the pair number eight); **D** – *Nycticeinops schlieffenii*, IVB S1378 (secondary constriction on the pair number 11); **E** – *Scotoecus hirundo*; IVB S1480, **F** – *Pipistrellus hesperidus*, IVB S592 (secondary constriction on the eleventh pair of chromosomes). The small black lines indicate the positions of the secondary constriction (even if not visible in both of the presented chromosomes of the respective pairs).

We obtained non-differentially stained karyotypes for 48 individuals assigned to six species, namely *Neoromicia somalica*, *N*. *rendalli*, *N*. *nana*, *Nycticeinops schlieffenii*, *Scotoecus hirundo* and *Pipistrellus hesperidus* (Table 1 and Additional file 1). For each species, the diploid number of chromosomes (2n), chromosomal arms number (FN), autosomal arms number (FNa) and morphology of chromosomes was recorded (Table 1). A secondary constriction was noted in all species except *Scotoecus hirundo*, while *Neoromicia somalica* was the only species with a bi-armed Y chromosome. The karyotypes of the species currently assigned to *Neoromicia* shared the number of arms (FN = 54, FNa = 50), but differed in the number of chromosomes. *Neoromicia somalica* (2n = 28, FN = 54, FNa = 50) had nine large metacentric and submetacentric, one medium-sized submetacentric, two small subtelocentric and one small acrocentric pairs of chromosomes. The X chromosome was a medium-sized metacentric, while the Y was a small submetacentric. A secondary constriction was observed on one pair (no. 7) of large submetacentric autosomes (Figure 1A). The chromosomal complement of *Neoromicia rendalli* (2n = 38, FN = 54, FNa = 50) contained six large metacentric and submetacentric, one small subtelocentric and eleven acrocentric pairs. The X chromosome was a medium-sized submetacentric, while the Y was dot-like (based on our subjective view, presumably acrocentric). A secondary constriction was recorded on a single pair of acrocentric chromosomes (no. 9; Figure 1B). *Neoromicia nana* (2n = 34, FN = 54, FNa = 50) had eight pairs of large bi-armed chromosomes (a large pair of submetacentric chromosomes had a conspicuous secondary constriction – no. 8), one pair was small submetacentric and the remaining seven pairs were small acrocentric. The X sex chromosome was a medium-sized subtelocentric, the Y was dot-like and probably acrocentric (Figure 1C). The analysis of *Nycticeinops schlieffenii* (2n = 34, FN = 56, FNa = 52) revealed seven metacentric and submetacentric, one medium large metacentric, two small metacentric and six acrocentric pairs of chromosomes. A distinct secondary constriction was situated on the largest acrocentric pair (no. 11). The X chromosome was a medium metacentric and the Y chromosome was dot-like in appearance (Figure 1D). The karyotype of *Scotoecus hirundo* (2n = 30, FN = 50, FNa = 46) consisted of six large metacentric and submetacentric, three medium-sized bi-armed (submetacentric and subtelocentric) and five acrocentric autosomal pairs of chromosomes. The X chromosome was a medium-sized metacentric and the Y dot-like, probably acrocentric (Figure 1E). The highest diploid number of chromosomes and chromosomal arms was found in *Pipistrellus hesperidus* (2n = 46, FN = 62, FNa = 58), which comprised three large metacentric, two smaller submetacentric, two small submetacentric, and 15 acrocentric pairs of chromosomes (one pair with a secondary constriction – no. 11). The X chromosome was a medium-sized metacentric and the Y chromosome was dot-like (Figure 1F).

**Table 1:** Synoptic list of the main karyotypic characteristics of the 6 species from Senegal and numbers of specimens examined

**Species**	**2n**	**FNa**	**FN**	**X**	**Y**	**Specimens analysed**
*Neoromicia nana*	34	50	54	ST	D (A)	9♂, 7♀
*Neoromicia rendalli*	38	50	54	SM	D (A)	2♂
*Neoromicia somalica*	28	50	54	M	SM	13♂, 7♀
*Nycticeinops schlieffenii*	34	52	56	M	D	4♂, 1♀
*Pipistrellus hesperidus*	46	58	62	M	D	3♂, 1♀
*Scotoecus hirundo*	30	46	50	M	D (A)	1♂

Abbreviation: Following characteristics are described: *2n* diploid number of chromosomes, *FNa* number of autosomal arms, *FN* number of chromosomal arms, *M* metacentric, *SM* submetacentric, *ST* subtelocentric, *A* acrocentric, *D* dot-like chromosome, *X*, *Y* morphology of the sex chromosomes, ♂ male, ♀ female. The brackets indicate the cases when the morphology of the Y chromosome could only be subjectively assumed.

### Molecular phylogenetic analyses

#### Single, mitochondrial and nuclear gene analyses

Based on the initial ML and BA analyses of the concatenated *cytb* and *tRNA*^*Thr*^ data from Senegal ([GenBank: JX276105–JX276317]; Additional file 1) and GenBank (Additional file 2; total number of taxa including GenBank data *n* = 361, sequence length = 1,212 bp), we found that the 213 individuals from Senegal represented 10 species assigned to *Myotis bocagii* (*n* = 1), *Neoromicia nana* (*n* = 98, 29 haplotypes), *N*. *somalica* (*n* = 93, 53 haplotypes), *N*. *capensis* (*n* = 1), *Pipistrellus hesperidus* (*n* = 6, 5 haplotypes), *Pipistrellus rueppellii* (*n* = 2, 2 haplotypes), *Scotoecus hirundo* (*n* = 1), *Neoromicia rendalli* (*n* = 2, 2 haplotypes), *Nycticeinops schlieffenii* (*n* = 8, 8 haplotypes) and *Glauconycteris variegata* (*n* = 1; phylogenetic tree in Additional file 3; haplotypes in Additional file 1). We calculated the *cytb* sequence variation using the Kimura two-parameter model of base substitution (K2P) in Phylip version 3.69 ([35]; Additional file 4A) and compared the results with the generally followed genetic criteria for delimiting taxa [36]. The variation between the respective specimens of each Senegalese taxon was less than 1.5% (Additional file 4A), which is in agreement with the typical intraspecific bat divergence (under 2.3% [36]).

Selected specimens representing 10 species were sequenced for six additional genes: *rag1* (20 individuals, [GenBank: JX276320–JX276339]), *rag2* (20 individuals, [GenBank: JX276340–JX276359]), *12S*, *tRNA*^*Val*^ and *16S* (18 individuals, [GenBank: JX276360–JX276377]), and *nd1* (2 individuals, [GenBank: JX276318–JX276319]; Additional file 1). These individuals were used in subsequent ML analyses of single genes and combined mitochondrial (*cytb* +*12S* + *tRNA*^*Val*^; ML, BA) and nuclear genes (*rag1* + *rag2*; ML) analyses. In the *12S* tree, *G*. *variegata* was sister to *G*. *beatrix* (bootstrap support BS = 86), and both these species were related to a clade consisting of *G*. *argentata* and *G*. *egeria* (BS = 100; Additional file 5A). Yet, in the nuclear gene tree (concatenated *rag1* and *rag2*), *Glauconycteris variegata* was basal to all *Glauconycteris* (BS = 100; Additional file 5C).

To test the influence of phylogenetic methods used on the position of *Pipistrellus rueppellii*, we reanalysed the *nd1* dataset (900 bp, *n* = 217; originally analysed with Neighbour-joining (NJ) and Kimura 2-parameter distances) of Mayer et al. [37] with ML and Bayesian methods. *Pipistrellus rueppellii* was basal (together with *P*. *nathusii*) to the *Pipistrellus*/*Nyctalus* clade (BS = 74, Bayesian posterior probabilities PP = 1; Additional file 6), a position similar to the phylogenetic analyses of the concatenated genes of this study (see results below).

### Multi-locus analysis of eight genes

**Figure 2:**
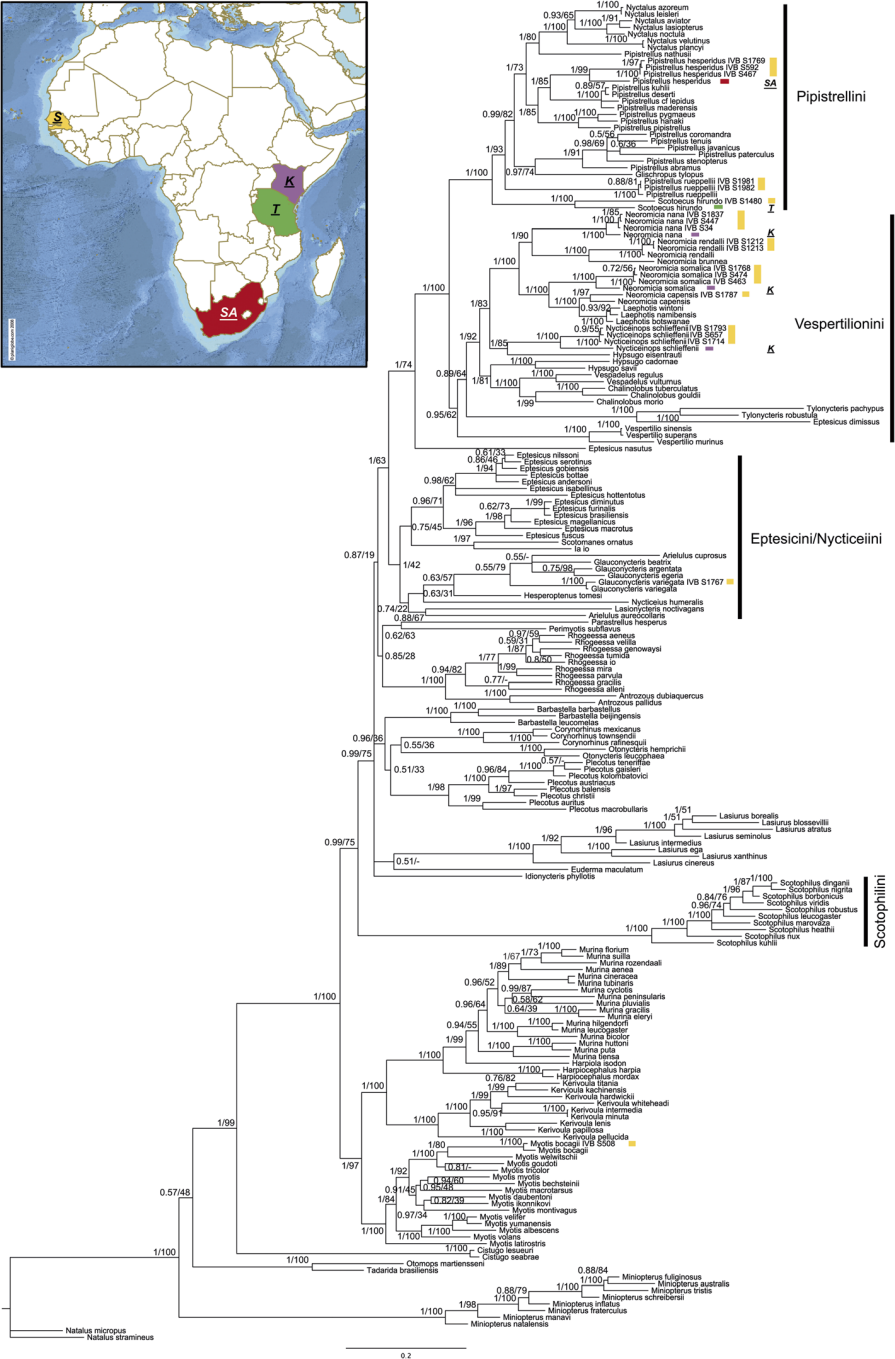
**Phylogenetic tree.** Phylogram of vespertilionid bat species from Senegal (IVB S) and GenBank data with selected tribes indicated. A Bayesian phylogenetic tree based on the concatenated dataset of 6 mitochondrial and 2 nuclear genes (*cytb* + *tRNA*^*Thr*^ + *12S* + *tRNA*^*Val*^ + *16S* + *nd1* + *rag1* + *rag2*; 5,665 bp; total *n* = 200) is presented. Nodes supports are indicated by posterior probabilities and/or bootstrap values resulting from ML analysis of the same dataset (BA values are left and ML values right of the hashes). Nodes, which were not supported with ML and were in different position than in BA, are indicated with “-”. Nodes are considered supported when Bayesian posterior probabilities are ≥0.95 and/or ML bootstrap proportions are ≥75%. The bar indicates genetic distance (the number of nucleotide substitutions per site). The map of Africa, shows the sampling localities of the five taxa considered to be cryptic in Senegal. The map shows the position of Senegal (***S***) and the countries, where the specimens from GenBank, used here for the genetic distance comparison, were sampled: ***SA*** – South Africa (*Pipistrellus hesperidus*), ***T*** – Tanzania (*Scotoecus hirundo*), ***K*** – Kenya (*Neoromicia somalica*, *Nycticeinops schlieffenii*, *Neoromicia nana*). The same symbols and colours are used to show the origin of the respective specimens in the phylogram.

The phylogenetic analyses of the combined dataset using ML and BA resulted in similar topologies (eight genes; 20 specimens of 10 Senegalese species; 180 taxa from GenBank; Accession numbers listed in Additional file 1 and Additional file 2). There was no node that was statistically supported in a different position in different analyses (the only exception was the weak support of *Glauconycteris*, see below). The Bayesian phylogram with proportional branch lengths and confidence for the nodes recovered by both phylogenetic methods (PP and BS) is presented (Figure 2, if not stated differently, all the following results refer to this figure and to both methods).

Five species from Senegal were not distinctive from individuals from other African localities (*Myotis bocogii*, *Neoromicia nana*, *N*. *rendalli*, *Pipistrellus rueppellii* and *Glauconycteris variegata*). However, the other five Senegalese taxa differed from their putative species – *Pipistrellus* cf. *hesperidus*, *Nycticeinops* cf. *schlieffenii*, *Scotoecus* cf. *hirundo*, *Neoromicia* cf. *nana* and *N*. cf. *somalica* (Figure 2). The genetic distance for *cytb* between *Pipistrellus* cf. *hesperidus*, *Neoromicia* cf. *nana* and *N*. cf. *somalica* and its respective “conspecific” from other populations was large 4.79-13.18% (Additional file 4A). No comparable *cytb* sequences were available for the two other taxa, but the differences for other genes were also large. *Nycticeinops* cf. *schlieffenii* from Senegal differed from its conspecifics by 5.56-5.91% for *12S*. The *12S* divergence between the remaining available taxa from Senegal and respective GenBank samples from other populations was about 1.87-6.26% for the cryptic taxa and less than 1% for the others (Additional file 4B). The genetic divergence between the Senegalese and the Tanzanian specimen of *Scotoecus hirundo* was 1.6% for *rag2* which is relatively high (the range of “non-cryptic” taxa was 0–0.68%; Additional file 4C).

Based on both Senegalese and GenBank data, we found support for the composition of the tribes Pipistrellini (*Nyctalus*, *Pipistrellus*, *Scotoecus* and *Glischropus*) and Vespertilionini (*Neoromicia*, *Laephotis*, *Hypsugo*, *Vespadelus*, *Nycticeinops*, *Tylonycteris*, *Eptesicus dimissus*, *Vespertilio* and *Chalinolobus*). In the Pipistrellini, where Senegalese specimens were also included in the analysis, *Pipistrellus rueppellii* was well supported in a basal position within the *Pipistrellus*/*Nyctalus* clade (PP = 1, BS = 93) and *Scotoecus hirundo* was basal to this whole group (PP = 1, BS = 100). *Pipistrellus hesperidus* appeared clearly distinct from its sister group containing *P*. *kuhlii* (PP = 1, BS = 85). The Asian species *Glischropus tylopus* (data only from GenBank) was included within the Pipistrellini, as basal to the clade of the East Asian species of *Pipistrellus* (*P*. *coromandra*, *P*. *tenuis*, *P*. *paterculus*, *P*. *stenopterus*, *P*. *javanicus* and *P*. *abramus*) with reasonable support (PP = 0.97, BS = 74). In the tribe Vespertilionini, the genus *Hypsugo* was polyphyletic. *Nycticeinops schlieffenii* (including individuals from Senegal) was sister to *Hypsugo eisentrauti* (PP = 1, BS = 85), while *H*. *cadornae* and *H*. *savii* formed a distinct group related to the *Chalinolobus* /*Vespadelus* clade (PP = 1, BS = 81). The analyses included west-African specimens belonging to several species assigned to *Neoromicia*. *Neoromicia nana* was related to *N*. *brunnea* and *N*. *rendalli* in single gene trees and in the trees based on the concatenated nuclear genes (*cytb*, Additional file 3; *rag1* + *rag2*, Additional file 5C). In the tree based on the eight genes, *Neoromicia nana* formed a clade with *N*. *brunnea* and *N*. *rendalli* (PP = 1, BS = 90), whereas, *N*. *somalica* and *N*. *capensis* showed close relationship to *Laephotis* (PP = 1, BS = 100; Figure 2).

In the tribe Eptesicini, one of the species occurring in Senegal, *Glauconycteris variegata* was found basal within the *Glauconycteris* clade only in the ML analysis (BS = 79). In the BA, the result was likely influenced by *Arielulus cuprosus*, which appeared in the *Glauconycteris* clade (unsupported). The genus *Eptesicus* (based only on published sequences) was polyphyletic. Both Asian *E*. *nasutus* and *E*. *dimissus* were clearly distinct from the *Eptesicus* clade. *Eptesicus dimissus* was sister and basal to *Tylonycteris* (PP = 1, BS = 100) within the tribe Vespertilionini; whereas *E*. *nasutus* was basal to both the clades Vespertilionini and Pipistrellini (PP = 1, BS = 74). *Nycticeius humeralis* (data only from GenBank) was one of the basal taxa of a clade that included *Glauconycteris*, *Hesperoptenus*, *Arielulus* and *Lasionycteris*. However, there was little support for the intra-relationships within this clade, sister to a clade that contained *Eptesicus* (except for *E*. *nasutus* and *E*. *dimissus*), *Scotomanes* and *Ia* (PP = 1, BS = 42).

The tribe Scotophilini was supported as the second most basal branch within the vespertilionid bats (PP = 0.99, BS = 75). In the Myotini, *Myotis bocagii* represented a sister branch to *M*. *welwitschii* within the Ethiopian clade of the genus *Myotis* (PP = 1, BS = 80).

## Discussion

Phylogenetic relationships of the family Vespertilionidae are an area of active research (e.g. [7-10,38]). Our results from West African populations have found evidence of cryptic taxa and clarified several phylogenetic relationships within the vespertilionids, as well as supported certain tribes contrary to some recent molecular taxonomic systematic discussions [10,12,39].

### Karyotypes

We found differences in the diploid chromosome numbers and/or in chromosome morphology between the populations from Senegal and other regions of Africa in four out of six bat species examined (*Neoromicia* cf. *somalica*, *N*. cf. *nana*, *Scotoecus* cf. *hirundo* and *Pipistrellus* cf. *hesperidus*) and divergence to at least some African populations in one (*Nycticeinops* cf. *schlieffenii*).

The karyotype of *N*. *somalica* from Senegal contained one additional pair of acrocentric autosomes compared to the *N*. *somalica* from Cameroon (2n = 26, FNa = 48 [40]). It also differed from the karyotype of *N*. *zuluensis* (previously included in *N*. *somalica*; see [2,41]) from southern Africa (2n = 28, FNa = 48 [41]) and South Africa (2n = 28, FNa = 50 [38]). Despite sharing the same diploid number with *N*. *zuluensis*, *N*. *somalica* had a different structure of some bi-armed chromosomes and the X chromosome was metacentric in *N*. *somalica*, while being subtelocentric in *N*. *zuluensis*[38,41]. Thus, this is the first finding of a karyotype with a metacentric X chromosome within the *N*. *somalica*/*zuluensis* complex. Additionally, from the populations examined in southern Africa, the Senegalese specimens differed in the number of acrocentric autosomes (one pair – this study; two pairs [41]).

The Senegalese specimens of *Neoromicia nana* lacked two pairs of acrocentric autosomes compared to previous findings, but the karyotype contained an additional metacentric pair, which could have arisen by a Robertsonian fusion of the two acrocentric pairs. The X chromosome was subtelocentric, while it was metacentric in other studies (2n = 36, FN = 50 [38,41,42]). The karyotype of *N*. *rendalli* from Senegal is very similar to those found in populations from Somalia [40], Zimbabwe [43] and Southern Africa [38]. The only difference was in the relative size and morphology of the sex chromosome X. In Senegal it was conspicuously smaller than in the Somalian and Zimbabwean specimens, and it was submetacentric, while in Southern Africa it was metacentric. The diploid chromosome numbers of *N*. *nana*, *N*. *brunnea* and *N*. *rendalli* range from 34 to 38, with a stable FNa = 50 (this study; [40]). However, other members of this clade are reported to have karyotypes with lower numbers of chromosomes: *N*. *capensis* – 2n = 32, FNa = 50 [4,38,41,42] and *Laephotis botswanae*, *namibensis* and *wintoni* – 2n = 34, FNa = 50 [41]. Thus, the non-differentially stained karyotypes were not helpful in distinguishing the deeper relationships within the clade detected by molecular data.

*Nycticeinops schlieffenii* found in Senegal was karyotypically similar to findings from Somalia [44], while being conspicuously different in the number of chromosomes and chromosomal arms from the Southern African populations (2n = 42, FNa = 50 [41]), thus confirming the existence of two distinct species in Africa [15]. The karyotype of *Scotoecus hirundo* from Senegal (2n = 30, FN = 50, FNa = 46) had a reduced number of arms than reported previously from the Ivory Coast (2n = 30, FN = 54, FNa = 50 [20]), thus indicating that the specimen from Senegal may also represent a distinct species.

All specimens from the Senegalese populations of *Pipistrellus hesperidus* had a higher diploid number of chromosomes (2n = 46, FNa = 58) than specimens from South Africa and Madagascar (2n = 42, FNa = 50 [4,38,41]). The three smallest chromosomal pairs of the Senegalese specimens that are additional to other karyotypes of *P*. *hesperidus* could be supernumerary B-chromosomes that consist of only heterochromatic material. If this was the case, the presence of B-chromosomes could be proved by banding methods (which was not possible here, see Material and Methods), or by discovering a specimen with an odd chromosome number. All specimens examined had 2n = 46 in all 5–10 cells that were analysed for each specimen. We are therefore confident that *P*. *hesperidus* from Senegal has a different karyotype.

The variability of chiropteran karyotypes occurs relatively rarely [41], which is in contrast with e.g. rodents and shrews [45]. Despite some taxa having conservative karyotypes, Vespertilionidae as a whole are quite karyotypically diverse (see review in [4,6,41]). It has been therefore hypothesized that each species may be characterized by a distinct karyotype [4]. The occurrence of intraspecific variability, as seen here in some species, supports the hypothesis that these taxa represent cryptic forms [4,41]. The role of chromosomal rearrangement in reducing gene flow between races has been an area of active research for decades (reviewed in [46]). Recent evidence suggests that gene flow is not always impeded between forms with different karyotypes [47]. However, the differences found between the karyotypes of the hypothetical cryptic taxa from Senegal and their nominal species are supported with other lines of evidence such as molecular sequence data and morphological characters (see also discussion below).

### Molecular phylogeny of Senegalese specimens

The Senegalese specimens of *Pipistrellus rueppellii* can be confidently assigned to *P*. *r*. *senegalensis*, which is distributed from Algeria to Senegal (type locality Richard-Toll in Senegal [2,32]). They were large (forearm length = 33.3, 35.8 mm) relative to other non West African races [48] and were genetically similar to Moroccan samples (*nd1*; [37]). However, contrary to previous results from Morocco [37] and Sardinia [49], the Senegalese *P*. *rueppellii* formed a well-supported long branch that was basal in the *Pipistrellus*/*Nyctalus* clade. We, therefore, re-analysed the complete published *nd1* data of Mayer et al. [37] with ML and BA and again recovered the basal position of *P*. *rueppellii*, confirming the differences between studies were due the different analytical approaches used (PP = 1, BS = 74; Additional file 6). The simple algorithm based methods such as neighbour joining used by Mayer et al. [37] may be biased by long-branch attraction [50]. Bayesian inference of phylogeny is especially useful in such cases, as it is capable of finding global rather than local optima during heuristic searches [51]. The discrepancy between our results and Veith et al. [49] is likely to be due to differences in the datasets as they used less taxa and only very short fragment of *16S* (different to ours; 560 bp).

The unique position of *P*. *rueppellii* within the tribe was also demonstrated by the large genetic distinctiveness to other related species. The *cytb* divergence to six Eurasian and one African *Pipistrellus* species was between 19.59-24.07% and to *Scotoecus hirundo* it was 22.71-26.33%. The inter-species variation of the other *Pipistrellus* species was between 4.19-23.29% (Additional file 4A). The mean observed genetic variation between sister taxa of *Pipistrellus* is 6.7% (3.3-14.7% in all bats) and for non-sister species (inter-generic) some 10.6-16.5% (8.4-15.7% for bats in general [36]). These divergences therefore support the hypothesis that *P*. *rueppellii* is a member of a distinct genus. In agreement with this finding, *P*. *rueppellii sensu stricto* has been previously classified in the subgenus *Vansonia*[52] and differences to other *Pipistrellus*-like bats were also noted in a recent morphological study [53]. Comparison with the type specimen of *P*. *vernayi* (synonym to *P*. *rueppellii*) from Maun, Ngamiland, Botswana [54], which is the type species of *Vansonia*[15], showed that the Botswanian sample had smaller ears (12 mm versus approximately 15 mm here) and tragus (4 mm versus 5.5 mm here). The other (cranial and external) measurements were similar. Only further sampling of specimens from the type country, Sudan (see [1]), and the type locality of *P*. *vernayi*[15], together with a full taxonomic treatment, will be able to resolve both the species and generic status of this species.

*Pipistrellus hesperidus* from Senegal was distinct from *P*. *kuhlii* as had been shown previously [4,38,55]. Three different subspecies, which may deserve elevation to species rank, are recognized in the *P*. *hesperidus* complex [1]: *hesperidus* (North-eastern Africa), *fuscatus* (Afro-tropical regions other than Southern Africa and Madagascar) and *subtilis* (Southern Africa and Madagascar). *Pipistrellus fuscatus* has also been recognized as synonymous to *hesperidus* with an additional subspecies *P*. *h*. *broomi* recognized in the populations from South Africa [15]. The Senegalese specimens of *P*. *hesperidus* were 13% divergent in *cytb* (Additional file 4A) to *P*. *hesperidus* from South Africa [13]. While it is not surprising that geographically distant populations are genetically divergent, divergence of this order is usually considered at least inter-specific [36]. The Senegalese *P*. cf. *hesperidus* therefore is likely to represent a distinct taxon from *P*. *hesperidus sensu stricto* (type locality Eritrea, Abyssinia, shores of the Red Sea [15]). However, without comparable material it is not possible to confirm if these samples represent one of the already named subspecies (see [1]) or a completely new species.

We confirm *Scotoecus* (*S*. *hirundo*) as sister to the *Pipistrellus*/*Nyctalus* clade [9,10] and therefore the validity of its inclusion in the tribe Pipistrellini, and not within Nycticeiini as previously thought [7,17,20]. Mitochondrial sequences from other *S*. *hirundo* are not available. However, the genetic divergence between the Senegalese and the Tanzanian specimen was 1.6% for *rag2* (Additional file 4C; [10]), which is relatively high. The uniqueness of the Senegalese specimens was also confirmed by the different karyotypes (compare with [20]). The specimens from both studies originated from Western Africa (Senegal, this study; Ivory Coast [20]), close to the type locality in Ghana [15]. Therefore, there are certainly two cryptic populations in West Africa, but more sampling is needed to determine which one represents the nominotypical species.

The polyphyly of *Neoromicia* and the separation of this genus into two lineages (the first containing *N*. *somalica*, *N*. *capensis* and *Laephotis* and the second containing *N*. *nana*, *N*. *brunnea* and *N*. *rendalli*) confirmed previous results [7,11,13]. Previously, *Laephotis* was found related to some *Neoromicia* species on the basis of bacular morphological characters [38]. Later, it was suggested to retain the name *Neoromicia* for *N*. *somalica* and to allocate *nana*, *brunnea* and *rendalli* to a separate, as yet unnamed, genus [7]. *Neoromicia nana* and *N*. *somalica* from Senegal were karyotypically distinct and also divergent in *cytb* (8-9% and 5%) from their respective conspecifics from Kenya [7]. These specimens are therefore likely to represent new taxa. Despite many revisions of the *Neoromicia* group (see review in [1,15]), the taxonomic status of the genus and species are still not successfully resolved and require further systematic work using samples from a wider geographic distribution.

*Nycticeinops schlieffenii* is distributed in three areas in Africa; the West, the East and South [15]. Molecular systematics separated the East and West populations at a species indicative level (more than 5% divergence for *12S*, this study; [7]), despite sharing similar karyotypes (this study; [44]). Moreover, southern populations have different karyotypes to the East/West populations (this study; [41,44]). The Eastern populations seem to be similar to the type from Egypt [15], but further taxonomic work is required to delimit the type’s distribution and correctly describe all the taxa. *Glauconycteris variegata* was supported in different positions within the genus based on the genes and/or methods used. Nuclear markers placed *G*. *variegata* as the basal species (from 4 used out of 12 described [1]); directly opposing a hypothesis based on the karyotypes where it was thought to be the derived state for the genus [56]. The basal position was only weakly supported (PP = 0.55, BS = 79) in the concatenated dataset (Figure 2). Phylogenetic analysis using a larger dataset placed *G*. *variegata* as sister to *G*. *argentata* and *G*. *egeria*, while *G*. *beatrix* was basal [9]. The sequences used here are all taken from Roehrs et al. [9] with only the addition of the Senegalese *G*. *variegata* that was not very variable from the Kenyan samples (Additional file 4B and Additional file 4C; [9]). Different taxon sampling or missing data could have caused the differences, but Roehrs’ result supported by a larger dataset (8,124 bp) is more probable.

### Molecular systematics of vespertilionids

The systematic relationships of vespertilionids revealed here were topologically similar to recent assessments (e.g. [7-10]); however, some phylogenetic positions were significantly different. We clarify the tribe containing *Glischropus*, two species previously assigned to *Eptesicus*, redefine the tribe Pipistrellini and Vespertilionini and clarify the position of the tribe Scotophilini. This tribe was demonstrated as basal to other vespertilionid bats based on *mt*DNA phylogenies (this study – Additional file 5A and Additional file 5B; [13,21,39]), but was supported as sister to Antrozoini using combined multi-locus datasets [9,10]. Our results place Scotophilini as the second most basal branch within all vespertilionids with reasonable support (PP = 0.99, BS = 75), similar to the phylogeny presented by Lack and Van Den Bussche [57]. The combined evidence from all recent studies confirms that Scotophilini are clearly distinct from other Vespertilionidae and deserve a full subfamily rank (Scotophilinae; or Philisinae, see Introduction and [15,21]).

*Glischropus tylopus* was significantly supported in Pipistrellini, within the clade of the East Asian pipistrelles. These results agree with cytogenetic data that placed *Glischropus* in Pipistrellini together with *Pipistrellus*, *Nyctalus* (and *Scotozous*, which is now excluded [4]), and previous molecular studies (unsupported [58]). The concatenated dataset confirmed the close relationship of *P*. *nathusii* to *Nyctalus* (PP = 1, BS = 80; Figure 2; [7,10]), in contrast to the most recent assessment (*16S*; [49]) and our results (*rag* genes or combined *cytb* and *tRNA*^*Thr*^ genes; Additional file 3 and Additional file 5C), where it appeared in other (even basal) positions within the *Pipistrellus*/*Nyctalus* clade. *Glischropus* with *Nyctalus* represent two genera that are morphologically distinguishable from *Pipistrellus*[59,60], but nest phylogenetically within this genus (this study; [7]). Systematically, this leaves *Pipistrellus* as diphyletic, which requires further investigation.

*Eptesicus dimissus* was related to *Tylonycteris* within the Vespertilionini and not to other *Eptesicus* species in Eptesicini. Previously, *Eptesicus dimissus* appeared either basal to *Pipistrellus* (maximum likelihood BS = 70, PP = 1.0), sister to *Hypsugo cadornae* (maximum parsimony BS = 90 [8]), or sister to *Tylonycteris*[57]. Here, it was strongly supported as sister to *Tylonycteris* (Figure 2). Likewise, *E*. *nasutus* was previously found distinct from the *Eptesicus* clade, but its exact position remained unsupported [61]. It has also been assigned to the subgenus *Rhineptesicus*[62], but this was later refuted [1,15]. Here, it was significantly distinct from *Eptesicus* and basal to Vespertilionini and Pipistrellini (PP = 1, BS = 74; Figure 2). *Eptesicus dimissus* and *E*. *nasutus* are rarely caught, but their phylogenetic distinctiveness to *Eptesicus* confirms this genus needs to be taxonomically revised.

Roehrs et al. [10] suggested using the name Vespertilionini for a tribe that only included *Nyctalus*, *Pipistrellus*, *Scotoecus* and *Vespertilio* (previously Pipistrellini). Here, the tribe *Vespertilionini* (as used by [5]) appeared well supported (*Vespertilio*, *Neoromicia*, *Hypsugo*, *Chalinolobus*, *Laephotis*, *Nycticeinops*, *Tylonycteris*, *Eptesicus dimissus* and *Vespadelus*). These taxa have been described as the hypsugine group [10], but were previously recognized as Vespertilionini [5,7]. Our evidence supports retaining the following names for the respective tribes, i.e. Pipistrellini for *Nyctalus*, *Pipistrellus*, *Scotoecus* and *Glischropus* (similarly as recognized by [1,4,7,60]; but excluding *Perimyotis* and *Parastrellus*) and Vespertilionini, i.e. *Vespertilio*, *Neoromicia*, *Hypsugo*, *Chalinolobus*, *Laephotis*, *Nycticeinops*, *Tylonycteris*, *Eptesicus dimissus* and *Vespadelus* (similarly as recognized by [7]).

*Nycticeius humeralis* appeared in a clade that contained members from the genera *Eptesicus*, *Scotomanes*, *Lasiurus*, *Arielulus*, *Glauconycteris*, *Hesperoptenus* and *Lasionycteris*. However, this position was supported only in the tree based on combined mitochondrial genes (Additional file 5B; PP = 0.95). If this topology (similar to [9]) approaches the truth, it would require the tribe to be recognized as Nycticeiini, rather than Eptesicini on the basis of priority [9]. These taxa form long branches and, short of increasing taxon sampling, it may be that more data via Next Generation sequencing will be necessary to clarify these deep relationships.

## Conclusions

Phylogenetic analysis of the vespertilionid bats from Senegal have highlighted how little is known about the bat fauna of the region. Five taxa different enough to be considered new (cryptic) species under the genetic [36] and phylogenetic species concepts were detected (*Pipistrellus* cf. *hesperidus*, *Nycticeinops* cf. *schlieffenii*, *Scotoecus* cf. *hirundo*, *Neoromicia* cf. *nana* and *N*. cf. *somalica*). Despite the known limitations of the genetic species concept [63], all these findings are additionally supported by karyotypical divergences. Clearly a full taxonomic assessment is required to determine if there are hidden morphological characters that would support the delimitation of these species. The presence of cryptic (morphologically similar, but genetically and phylogenetically distinct) taxa pairs across the tree of Vespertilionidae suggests a signal of long genetic isolation between West- and other African populations (probably about 0.4-6 million years ago at species level, compare with [13,64]). The studies of past climatic oscillations in subtropical Africa are not numerous, but there is good evidence of periodic shifts from drier to more humid conditions during the Pliocene-Pleistocene transition, at the same time as shifts between glacial and moderate climate conditions at higher latitudes [65]. One of the arid periods and subsequent decrease of tree cover (1.8-1.6 Mya) is evidently related to taxonomic diversifications at species level in some western and central African forest pteropodids, while the intraspecific divergences based on standard mammalian rate of evolution probably appeared at the end of Pleistocene (about 0.1-0.4 Mya [64]). This is similar for the African *Myotis*, where population divergence is recent (Pleistocene), but species diversification occurred earlier, at about 6 Mya [13] when aridity caused massive steppe expansion across tropical Africa [66]. Late Pleistocene and Holocene refugia have been hypothesized along the Atlantic coast of western Africa for several west and central African plants and rodents [67,68]. Similar refugia have been detected in eastern, central and southern parts of Africa, for both small and large mammals inhabiting forest or savannah [69]. Three Senegalese taxa could be roughly dated from their conspecifics (based on the mean mammalian *cytb* sequence evolution rate adjusted for bats to about 0.02-0.05 per lineage per 1 million years and results of other works on African bats [13,64]). The split between *Neoromicia* cf. *somalica* and *N*. cf. *nana* from Senegal and respective populations from east Africa dated to 3.2 Mya and *Pipistrellus* cf. *hesperidus* split from South African populations at around 4.2 Mya. The divergence of *P*. *hesperidus* would therefore have occurred sometime during the Pliocene’s long trend of forest expansion while the *Neoromicia* species divergence corresponds with the time known as the onset of a dramatic tree cover decline in the region [66]. One of the well-established biogeographic barriers responding to the fluctuations of past climate changes between the rainforests of East and West Africa is the so-called Dahomey Gap [68]. Today it is some 200 km wide, but is known to have closed and opened through time (probably in relation to the arid/humid periods [70,71]). This savannah corridor represented a dispersal barrier for many forest-dwelling species [70,72,73], but mammalian and bird species inhabiting savannahs and more arid regions have also shown high genetic differentiation between north/west and south/east African populations [74-76]. Bats are hypothesized to be able to cross the Dahomey Gap [64,77,78]; but establishing populations within the gap would be unlikely for forest-dwelling species with small ranges [64,79]. Moreover, the Niger Delta or the Cameroon volcanic line have also been hypothesised to act as environmental barriers for some pteropodid populations from West and Central Africa [64]. There are few data across Africa to truly assess distributions and habitat type preference of the species examined here [1,15,29] and without denser sampling we are really limited in the conclusions that can be drawn. The mechanism determining speciation in each species will depend on many factors, including ecology, flight abilities and home-range fidelity, as well as exposure to abiotic, vicariant or climatic events. Further studies are therefore required to understand how population ecology and dispersion contribute to gene flow and to the diversification of vespertilionids in the region.

West Africa (especially the forest zone, up to the Nigeria/Cameroon boarders) is recognised as a region with high number of endemic organisms and as among one of the most fruitful parts of the continent for describing new cryptic taxa [80]. Recently, new species have been found within rodents, reptiles and insects [73,81,82]. Likewise, cryptic forms and new chromosomal races were detected in Senegal and other parts of Western Africa in pteropodid, rhinolophid, hipposiderid and vespertilionid bats (this study; [11,64,83-85]). This makes this area a hotspot for further chiropteran species discovery, and is in agreement with a general view that West Africa is one of the world's key hotspots of biodiversity [28].

New taxa and new systematic arrangements together with support from karyotypes demonstrate that there is still much to uncover in the vespertilionids and that morphologically similar species that occur in Africa are a result of convergent evolution and belong to phylogenetically distant groups. Morphologically distinct species can be ecologically and even genetically very similar and *vice versa* – closely-related species may be morphologically undistinguishable while being different in ecology and echolocation calls, as a consequence of convergent adaptive evolution [86,87]. Cryptic taxa with little morphological characteristics are an increasingly common phenomenon in *Pipistrellus*-like bats and genetic data are the most powerful tool for distinguishing them reliably [36,86]. Considering the frequent occurrence of hybridization and introgression in rapidly evolving lineages, including bat species of this group [88], both mitochondrial and nuclear data are necessary to make phylogenetic inference on historical relationships. While there is still a taxonomic impediment to provide keys and aid ecological work on these species, molecular systematics in conjunction with an independent character state such as karyotypes has confirmed several taxa as distinct and greatly adds to our understanding of biogeographic history of this diverse group of bats.

## Material and methods

### Sampling and localities

**Figure 3:**
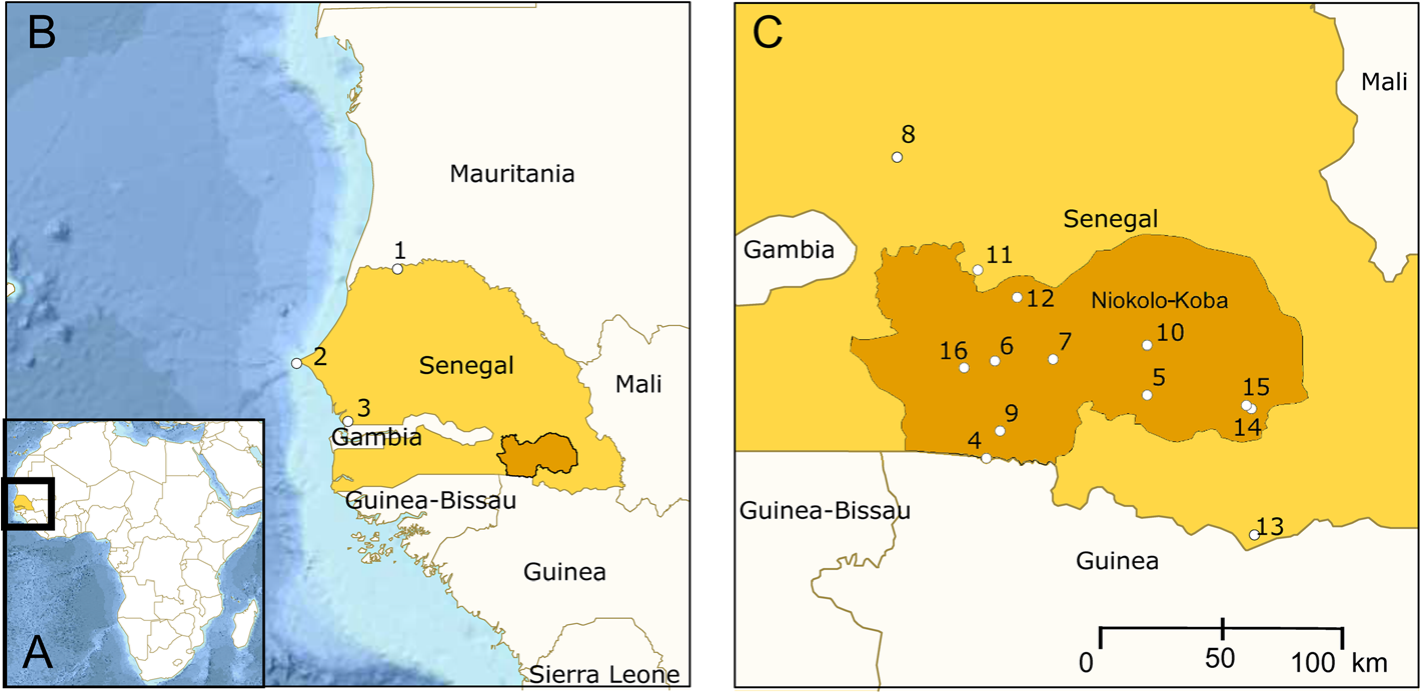
**Sampling localities. A** – map of Africa, bold square indicates the sampling area in West Africa; **B** – map of Senegal and Gambia with position of the area of the Niokolo-Koba National park (orange) and sampling localities: 1 – Mbilor, 2 – Hahn-Dakar, 3 – Bacadadgi; **C** – Niokolo-Koba National Park (orange): 4 – Gué de Sambaillou, 5 – Mont Assirik, 6 – Simenti + Camps de Lions, 7 – Lengué Kountou, 8 – Tambacounda + Tambacounda-Parc National de Niokolo-Koba, 9 – Dalaba, 10 – Niokolo, 11 – Niériko + Niériko-bridge, 12 – Dar Salam, 13 – Dindéfélo + Dindéfélo II, 14 – Mako-Camp, 15 – Mako, 16 – Gué de Damantan. The map was created with Planiglobe [89]. See Additional file 1 for coordinates of each locality.

Two hundred and thirteen vespertilionid bats were obtained during seven expeditions to Senegal (Western Africa) between 2004–2008 from 20 collection sites mainly in the Niokolo-Koba National Park (Figure 3; Additional file 1). Collection sites and timing of expeditions were chosen to maximize the diversity of vespertilionid communities sampled throughout the year. Preliminary species determinations were based on morphological characters using the keys of Kingdon [59] and Rosevear [29]. Standard external body measurements were taken using callipers and the body mass was measured using a spring scale (data shown only for *Pipistrellus rueppellii*). The nomenclature generally followed Simmons [1], unless revised by subsequent taxonomic assessment (e.g. [8-10,15]). Chromosome preparations were completed in 48 specimens. Biological material of voucher specimens (skulls, skins, tissue samples for molecular analyses or whole animals), as well as the chromosome slides are deposited in the Institute of Vertebrate Biology (IVB), Academy of Sciences of the Czech Republic, Brno, Czech Republic. Sampling in Senegal was approved by the Senegal’s National Parks General Management, Dakar (Specimens are listed in Additional file 1).

### Cytogenetics

Chromosome preparations were completed in the field following the bone marrow direct method modified after Baker [90]. The standard non-differential Giemsa staining was performed. Attempts to apply differential staining failed, probably because of ageing of the slides that were prepared and stored in field conditions. Digital microimages of the metaphases were taken with the Zeiss AxioCam MRm or Olympus DP 30Win cameras attached to the Provis AX 70 Olympus microscope. Karyotypes were assembled with the software for cytogenetic analysis Ikaros (MetaSystems GmbH, Germany) and final figures prepared with Corel PHOTO-PAINT 12 or X3 (Corel Corporation). The classification of chromosomes according to the position of the centromere followed published criteria [91].

### DNA extraction, PCR and sequencing

Total genomic DNA was extracted from tissue samples stored in alcohol with DNeasy Blood & Tissue Kit (Qiagen) or *forensic*GEM Saliva (ZyGEM). The genes were amplified by the polymerase chain reaction (PCR). Each 25 μl reaction cocktail contained 12.5 μl of PPP Master Mix (2.5 U *Taq* purple DNA polymerase, 200 μM of each dNTPs, 75 mM Tris–HCl, pH 8.8, 20 mM, (NH_4_)_2_SO_4_, 0.01% Tween 20), 1.5 μl 2.5 mM of MgCl_2_ and 8 μl of PCR water (all from Top-Bio), 1 μl of each primer (0.4 μM) and 1 μl of template DNA (all extracted DNA samples were diluted to a standardized concentration 2 ng/μl). A specific primer set was designed for the joint amplification of *cytb* and *tRNA*^*Thr*^ genes whereas published primer sets were used for the joint amplification of *12S*, *tRNA*^*Val*^ and *16S*[92] and for *rag1* and *rag2* genes ([93]; Table 2). Additionally, the *nd1* gene was sequenced from two individuals of *Pipistrellus rueppellii* using the PCR protocol and primers from Mayer et al. ([37]; Table 2), in order to test the genetic divergence of the Senegalese specimens from other populations. PCRs for all genes were performed as follows: initial denaturation for 2 min at 95°C, denaturation for 30 s at 94°C, annealing for 60 s, extension for 90 s at 72°C and final extension for 10 min at 72°C. The number of cycles and annealing temperatures of respective primers are reported in Table 2.

**Table 2:** Primers and details of PCRs used in the study

**Gene(s)**	**Primer name**	**Primer sequence**	**Annealing temperatures and no. of cycles**	**Reference**
*cytb* and *tRNA*^*Thr*^	DK14169F	5′-CCACGACTARTGACACGAAAAATC-3′	touchdown from 60 to 56°C, then 55°C for 30 cycles	this study
	DK15396R	5′-CAGCTTTGGGTGTTGATGGT-3′		this study
*12S*, *tRNA*^*Val*^, *16S*	12C	5′-AAAGCAAARCACTGAAAATG-3′	50°C, 36 cycles	[92]
	12G	5′- TTTCATCTTTTCCTTGCGGTAC-3′		[92]
*rag1*	RAG1F1705	5′-GCTTTGATGGACATGGAAGAAGACAT-3′	touchdown from 60 to 58°C, then 57°C for 33 cycles	[93]
	RAG1R2864	5′-GAGCCATCCCTCTCAATAATTTCAGG-3′		[93]
*rag2*	RAG2F220	5′-GATTCCTGCTA(CT)CT(TC)CCTCCTCT-3′	touchdown from 60 to 58°C, then 57°C for 33 cycles	[93]
	RAG2R995	5′-CCCATGTTGCTTCCAAACCATA-3′		[93]
*nd1*	ND1F2	5′-GGCAGAGACCGGTAATTGCATAA-3′	52°C, 30 cycles	[37]
	ND1R	5′-GTATGGGCCCGATAGCTT-3′		[37]

PCR products were purified using Qiagen’s QIAquick PCR Purification or Minielute kits or PCRExtract mini kit (5PRIME). The samples requiring gel extraction were cleaned with QIAquick or Minielute Gel Extraction kits from Qiagen. All samples were sequenced in both directions with the same PCR primers using BigDye Terminator sequencing chemistry (Applied Biosystems) on ABI sequencers. Sequences were assembled, checked by eye and edited in Geneious Pro version 5 ([94]; see Additional file 1 for GenBank Accession numbers). Additional sequences that represented all available vespertilionid and selected outgroup taxa were downloaded from GenBank (*n* = 180 taxa; Additional file 2). For some species, not all the genes were available from the same specimen; therefore, data of two or more individuals were used (Additional file 2). In the case of the genus *Myotis*, a reduced dataset was chosen to represent the main clades following Hoofer and Van Den Bussche [7].

### Phylogenetic analyses

Nucleotide sequence alignment was performed using MAFFT version 5 [95] implemented in Geneious Pro version 5 [94]. The sequences of *12S*, *tRNA*^*Val*^ and *16S* were each aligned in MAFFT version 6 (online at [96]), using the Q-INS-i method that takes into account the secondary structure of the ribosomal RNA. The resulting alignments were checked visually and manually corrected. The alignments of *12S*, *tRNA*^*Val*^ and *16S* sequences were compared with the alignment of Lack et al. [8] and ambiguous gap regions were removed, in order to prevent misidentification of homology and/or avoid overweighting of a single evolutionary event [97,98]. Coding gene sequences were translated to amino acids to confirm that they are not nuclear pseudogenes (which would contain frame shifts or premature stop codons).

To re-evaluate the morphological assignment of the Senegalese specimens, all 213 were sequenced for *cytb* (1,140 base pairs) and of these 209 were also successfully sequenced for the *tRNA*^*Thr*^ (72 bp; Additional file 1). These data were supplemented with 148 other sequences of *cytb* and 11 of *tRNA*^*Thr*^ from GenBank representing 148 taxa (*n* = 361, 1,212 bp; Additional file 2). (The length of the dataset is the maximum length available. Some sequences were shorter; represented by partial sequence data, or several taxa lacked genes entirely. The data matrix is represented in Additional file 1 and Additional file 2, which show the genes used for each taxon/specimen). These initial phylogenetic analyses of the concatenated data were performed by partitioning each gene and the 3^rd^ codon of *cytb* to account for different rates of evolution (Additional file 3). For this and all subsequent analyses, representatives of the families Cistugonidae, Miniopteridae, Molossidae and Natalidae were used to ensure adequate taxon sampling. For each phylogenetic analysis the outgroup was explicitly defined *a priori* (usually *Natalus stramineus* if available).

Bayesian phylogenetic analyses were performed using non-parallel and parallel versions 3.1.2 and 3.2.0 of MrBayes software [51]. The GTR + I + Γ model was used for the Bayesian analysis and was selected for each gene dataset using the Akaike Information Criterion implemented in MrModeltest v2.3 [99]. Bayesian posterior probabilities were estimated using MCMC sampling in MrBayes. Two independent searches were performed, each set consisted of one cold and three heated chains (the temperature was left to default 0.2 in version 3.1.2 and 0.1 in 3.2.0), trees were sampled every 1000 generations and starting trees were random. The searches were set to stop when the average standard deviation of split frequencies decreased below 0.01, burn-in value was 0.25 of all samples. The remaining trees were used to construct a 50% majority rule consensus tree.

Maximum likelihood analysis was performed with RAxML v 7.3.0 (Randomized Axelerated ML [100]) on the CIPRES Science Gateway [101] using the following settings: substitution model – GTR + Γ, 500 rapid bootstrap replicates, bootstrapping and ML search for the best-scoring tree performed in a single run. The automated stop was used initially to test the number of bootstraps required [102]. Conversion was reached at 250 bootstrap replicates, so we therefore conservatively choose to rerun the analysis with 500 bootstrap replicates.

Based on the initial BA and ML trees, representative specimens from the Senegalese populations were chosen (a maximum of up to three per species) to sequence four additional mitochondrial and two nuclear genes. We sequenced 20 individuals for *rag1* and *rag2* and 18 of the same individuals for *12S*, *tRNA*^*Val*^ and *16S*. Repeated attempts to sequence two individuals for *12S*, *tRNA*^*Val*^ and *16S* failed. We also sequenced two specimens of *Pipistrellus rueppellii* for *nd1* to confirm their species assignment with available GenBank data (Additional file 1).

RAxML analyses were used to construct single gene trees to assess if there was any gene conflict [103]. The sequences of each gene were trimmed to the same lengths, single triplet insertions were removed from *rag* sequences and only representatives with all respective genes were used to minimize the effect of missing data. We also compared combined selected mitochondrial gene phylogenies (*cytb* +*12S* + *tRNA*^*Val*^; ML and BA) versus a ML phylogeny based on nuclear genes (*rag1* + *rag2*; for information on sequence lengths and *n* of taxa and for selected subtrees see Additional files 5A-C). In the single gene and combined analysis, we partitioned the 3^rd^ codon of *cytb* and *nd1*, and each gene (if applicable). Subsequently, we concatenated all eight genes from 200 samples (20 specimens from Senegal, 180 taxa from GenBank – Additional file 2). In some cases, sequences of more individuals had to be combined. We increased taxon sampling even if this meant including taxa that had partial gene sequences or were missing data and only the ambiguous gap regions in *12S*, *tRNA*^*Val*^ and *16S* were removed. The missing data were treated as real missing data (not as unknown nucleotides, or gaps). The single gene datasets comprised maximally of the following lengths: *cytb* – 1,140 bp, *tRNA*^*Thr*^ – 71 bp, *12S* – 920 bp, *tRNA*^*Val*^ – 64 bp, *16S* – 82 bp, *rag1* – 1,123 bp, *rag2* – 1,308 bp, *nd1* – 957 bp. The final length of the concatenated dataset was maximally 5,665 bp. The BA and ML analyses were completed as previously outlined, partitioning each gene and the 3^rd^ codon of *cytb* and *nd1* (Figure 2).

Additionally, we ran ML and BA analyses (partitioning the 3^rd^ codon) on the 217 *nd1* gene sequences (900 bp) originally used by Mayer et al. [37] to test the influence of methodology, specifically on the phylogenetic position of *P*. *rueppellii* (Additional file 6).

Pairwise genetic distances between sequences were computed for *cytb*, *12S* and *rag2* using Kimura two-parameter model of base substitution implemented in Phylip version 3.69 [35] using the default settings (program Dnadist; Additional file 4A-C).

### Availability of supporting data

The data sets supporting the results of this article are included within the article (and its additional files).

## Competing interests

The authors declare that they have no competing interests.

## Authors’ contributions

DK carried out the molecular genetic studies, karyotypic analyses, sequence alignment, phylogenetic analyses, participated in the design of the study, prepared all the tables and figures and wrote the manuscript. NI designed the molecular and phylogenetic parts of the study, coordinated the molecular laboratory works and phylogenetic computations, helped to interpret the data and draft the manuscript. PH participated in the design of the molecular and phylogenetic parts of the study and helped to draft the manuscript. PK managed the whole project, participated in the design of this study, collected the samples in the field, coordinated the preparation of chromosomal slides and organised specimen identifications from taxonomic experts. JZ designed the whole study, participated in the karyotype analyses and helped to draft the manuscript. All authors read and approved the final manuscript.

## Supplementary Material

Additional file 1 **Phylogenetic tree to identify the 213 bats from Senegal marked by their IVB number.** Bayesian phylogram presented based on the concatenated dataset of *cytb* and *tRNA*^*Thr*^ genes with the addition of species from GenBank (1,212 bp; number of all taxa *n* = 361). The Bayesian analysis was run partitioned for genes and the 3^rd^ codon position for *cytb*, PP are indicated for each node. ML analysis was run on the same partitioned dataset, using RAxML GTR + Γ model, bootstrap support values are indicated for each node. Nodes are considered supported when Bayesian posterior probabilities are ≥0.95 and/or ML bootstrap proportions are ≥75%. BA values are left and ML values right of the hashes. Click here for file

Additional file 2 **List of nucleotide sequences obtained from GenBank used in this study.** The table specifies the specimens and sequences used for each species. In some cases, data of two or more individuals were used to represent the respective species, because not all the genes were available from the same specimen. The table does not contain all the 217 specimens analysed for the *nd1* gene by Mayer et al. [37]. See their publication for the comprehensive list of these specimens. Here, we list only those sequences from their study used in the analyses, which also contained our data. The list of full references is at the bottom of the table. Click here for file

Additional file 3 **Tables of pairwise Kimura-2-parameter distances between sequences of selected genes of the specimens examined.** Values represent percentage of different bases between sequences. Table A – Distances between the *cytb* gene sequences of 361 specimens (213 from Senegal and 148 from GenBank). Table B – Distances between the *12S* gene sequences of 144 specimens (18 from Senegal and 126 from GenBank). Table C – Distances between the *rag2* gene sequences of 140 specimens (20 from Senegal and 120 from GenBank). See Legends in the respective tables explaining the meaning of colours used. Click here for file

Additional file 4 **List of specimens collected in Senegal with sampling localities and GenBank Accession numbers of all sequences used.** Asterisks indicate cases, in which the sequences for *tRNA*^*Thr*^ were not obtained (and only the *cytb* gene was analysed). The acronym IVB used in specimens' numbers stands for the Institute of Vertebrate Biology, Academy of Sciences of the Czech Republic, Brno, Czech Republic, where the samples are deposited. The letter S was assigned to the specimens originating from Senegal. The seventh column indicates the specimens for which karyotypes were obtained and gives the diploid number of chromosomes (2n). Click here for file

Additional file 5 **Subtrees of phylogenetic trees based on single, mitochondrial and nuclear datasets showing phylogenetic positions differing from the eight-gene-tree.** A – Subtree of the *12S* gene tree representing 18 selected specimens from Senegal and GenBank data (920 bp, *n* = 144), showing the position of *Scotophilus* and the relationships within the *Glauconycteris* clade. B – Subtree of the Bayesian concatenated *cytb* + *12S* + *tRNA*^*Val*^ genes tree of 18 specimens from Senegal and GenBank data (2,119 bp, *n* = 119) showing the position of *Scotophilus* and *Nycticeius humeralis*. C – Maximum likelihood concatenated *rag1* and *rag2* genes tree for 20 selected specimens from Senegal and GenBank data (1,832 bp, *n* = 140) showing the position of *Neoromicia* and the relationships within the *Glauconycteris* clade. Nodes are considered supported when Bayesian posterior probabilities are ≥0.95 and/or ML bootstrap proportions are ≥75%. BA values are left and ML values right of the hashes. Click here for file

Additional file 6 **Part of the Bayesian *nd1* gene tree based on data of Mayer et al. ([37]; 900 bp, *n* = 217).** Subtree showing the position of *Pipistrellus rueppellii*. Both ML and BA were run partitioned for the 3^rd^ codon. ML analysis was run using RAxML, GTR + Γ model. Nodes are considered supported when Bayesian posterior probabilities are ≥0.95 and/or ML bootstrap proportions are ≥75%. BA values are left and ML values right of the hashes. Click here for file
