# Correction: Hidden diversity in Senegalese bats and associated findings in the systematics of the family Vespertilionidae

**DOI:** 10.1186/s12983-026-00609-2

**Published:** 2026-04-10

**Authors:** Darina Koubínová, Nancy Irwin, Pavel Hulva, Petr Koubek, Jan Zima

**Affiliations:** 1 Department of Zoology, Faculty of Science, Charles University, Viničná 7, 12844 Praha 2, Czech Republic; 2 Biology Department, University of York, HeslingtonYork, YO10 5DD UK; 3 Institute of Vertebrate Biology, Academy of Sciences of the Czech Republic, Květná 8, 60365 Brno, Czech Republic; 4 Department of Forest Protection and Game Management, Faculty of Forestry and Wood Sciences, Czech University of Life Sciences, Kamýcká 1176, 16521 Prague-Suchdol, Czech Republic

**Correction: Frontiers in Zoology 2013, 10:48** 10.1186/1742-9994-10-48

Following publication of the original article [1], the authors reported an error in the Electronic supplementary materials. The downloadable files of additional file 1, 3 and 4 are not matched with their captions.

“Additional file 1: Phylogenetic tree to identify the 213 bats from Senegal marked by their IVB number” should be linked to **12983_2013_262_MOESM4_ESM.tiff**.

“Additional file 3: Tables of pairwise Kimura-2-parameter distances between sequences of selected genes of the specimens examined” should be linked to **12983_2013_262_MOESM1_ESM.xlsx**.

“Additional file 4: List of specimens collected in Senegal with sampling localities and GenBank Accession numbers of all sequences used” should be linked to **12983_2013_262_MOESM3_ESM.xls**.

## References

[1] Koubínová D, Irwin N, Hulva P, et al. Hidden diversity in Senegalese bats and associated findings in the systematics of the family Vespertilionidae. Front Zool. 2013;10:48. 10.1186/1742-9994-10-48. 23938084 10.1186/1742-9994-10-48 PMC3751436

