# Supplementary figures and images for: Hidden diversity in Senegalese bats and associated findings in the systematics of the family Vespertilionidae
Source: Front Zool. 2013 Aug 12;10:48. doi: 10.1186/1742-9994-10-48 (PMC3751436; doi:10.1186/1742-9994-10-48)

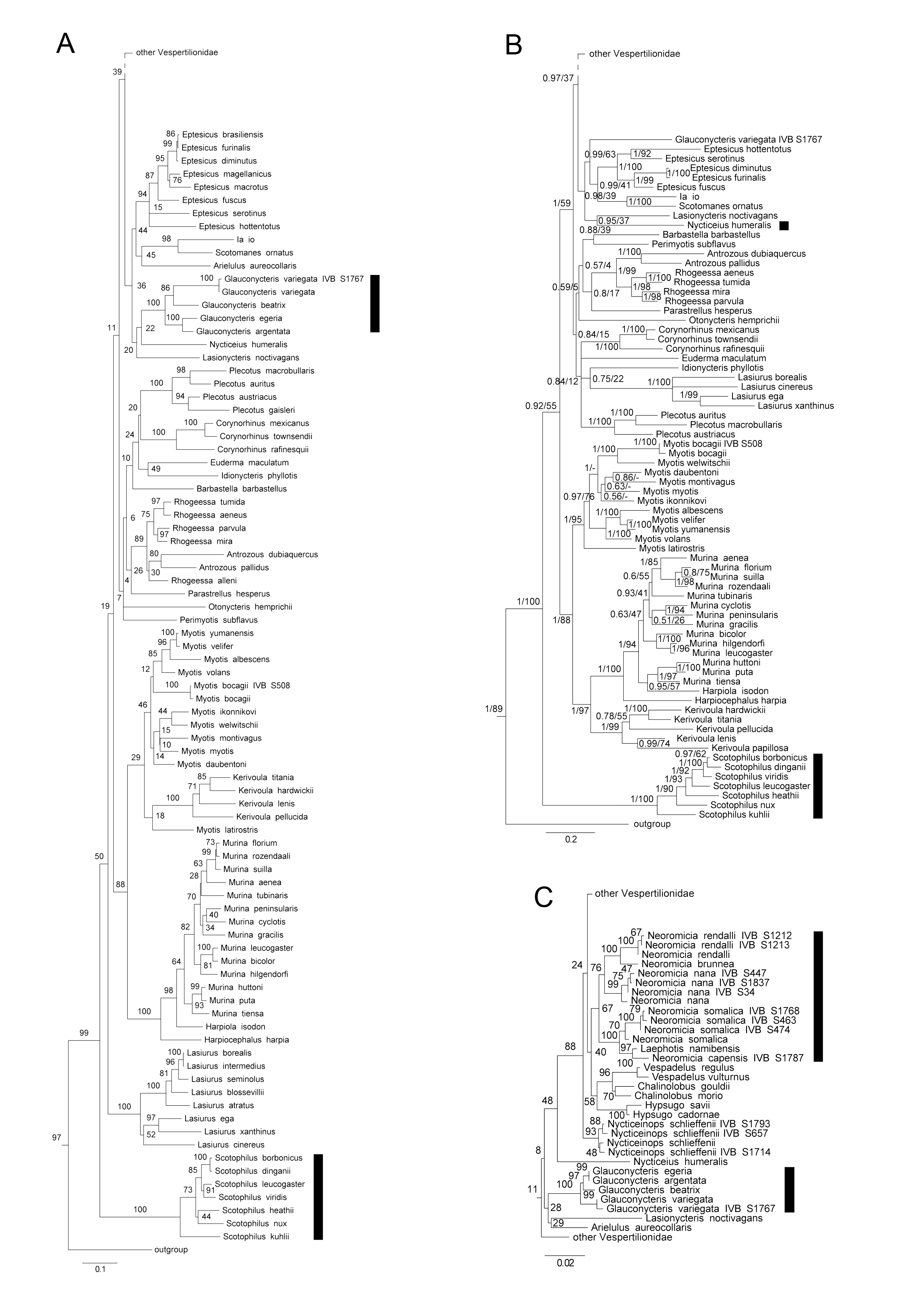

Supplement: Additional file 5 — Subtrees of phylogenetic trees based on single, mitochondrial and nuclear datasets showing phylogenetic positions differing from the eight-gene-tree. A – Subtree of the 12S gene tree representing 18 selected specimens from Senegal and GenBank data (920 bp, n = 144), showing the position of Scotophilus and the relationships within the Glauconycteris clade. B – Subtree of the Bayesian concatenated cytb + 12S + tRNAVal genes tree of 18 specimens from Senegal and GenBank data (2,119 bp, n = 119) showing the position of Scotophilus and Nycticeius humeralis. C – Maximum likelihood concatenated rag1 and rag2 genes tree for 20 selected specimens from Senegal and GenBank data (1,832 bp, n = 140) showing the position of Neoromicia and the relationships within the Glauconycteris clade. Nodes are considered supported when Bayesian posterior probabilities are ≥0.95 and/or ML bootstrap proportions are ≥75%. BA values are left and ML values right of the hashes. [file 1742-9994-10-48-S5.tiff]

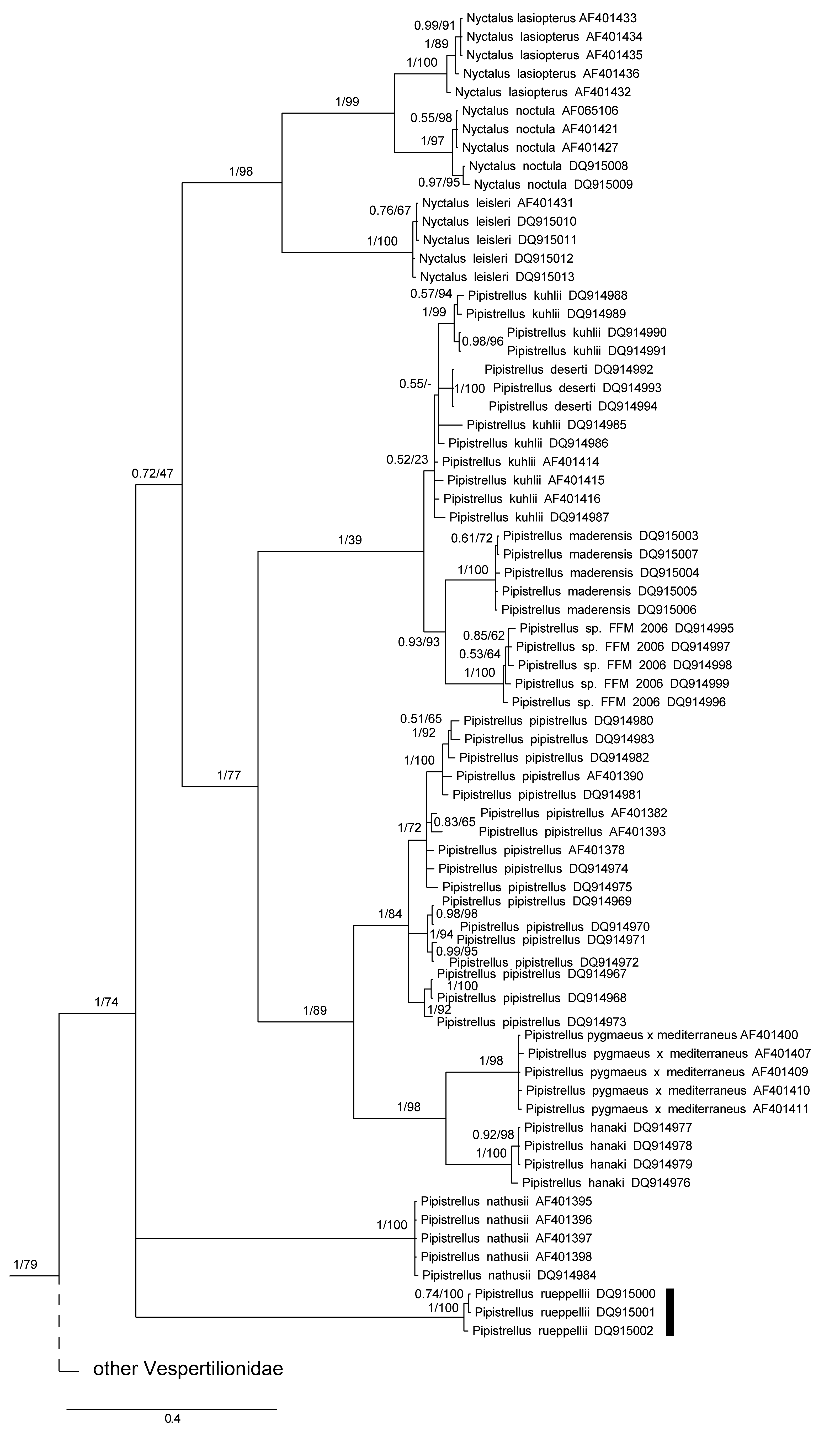

Supplement: Additional file 6 — Part of the Bayesian nd1 gene tree based on data of Mayer et al. ([37]; 900 bp, n = 217). Subtree showing the position of Pipistrellus rueppellii. Both ML and BA were run partitioned for the 3rd codon. ML analysis was run using RAxML, GTR + Γ model. Nodes are considered supported when Bayesian posterior probabilities are ≥0.95 and/or ML bootstrap proportions are ≥75%. BA values are left and ML values right of the hashes. [file 1742-9994-10-48-S6.tiff]
